# Epidemiology of West Nile Virus in the Eastern Mediterranean region: A systematic review

**DOI:** 10.1371/journal.pntd.0007081

**Published:** 2019-01-29

**Authors:** Sana Eybpoosh, Mehdi Fazlalipour, Vahid Baniasadi, Mohammad Hassan Pouriayevali, Farzin Sadeghi, Abbas Ahmadi Vasmehjani, Mohammad Hadi Karbalaie Niya, Roger Hewson, Mostafa Salehi-Vaziri

**Affiliations:** 1 Department of Epidemiology and Biostatistics, Research Centre for Emerging and Reemerging infectious diseases, Pasteur Institute of Iran, Tehran, Iran; 2 Department of Arboviruses and Viral Hemorrhagic Fevers (National Reference Laboratory), Pasteur Institute of Iran, Tehran, Iran; 3 Cellular and Molecular biology research centre, health research institute, Babol University of Medical Sciences, Babol, Iran; 4 Department of Virology, School of Public Health, Tehran University of Medical Sciences, Tehran, Iran; 5 Institute of immunology and infectious diseases, Iran university of medical sciences, Tehran, Iran; 6 Virology & Pathogenesis, National Infection Service, Public Health England, United Kingdom; 7 Research Centre for Emerging and Reemerging infectious diseases, Pasteur Institute of Iran, Tehran, Iran; Vienna, AUSTRIA

## Abstract

**Background:**

West Nile Virus (WNV), a member of the genus *Flavivirus*, is one of the most widely distributed arboviruses in the world. Despite some evidence for circulation of WNV in countries summarized by the World Health Organization as the Eastern Mediterrian Regional Office (EMRO), comprehensive knowledge about its epidemiology remains largely unknown. This study aims to provide a concise review of the published literature on WNV infections in the Eastern Mediterranean Regional Office of WHO (EMRO).

**Methodology/principal findings:**

A systematic review of WNV prevalence studies on humans, animals and vectors in the EMRO region was performed by searching: Web of Science, Science Direct, Scopus, PubMed, Embase and Google Scholar. Finally, 77 citations were included, comprising 35 seroprevalence studies on general population (24460 individuals), 15 prevalence studies among patients (3439 individuals), 22 seroprevalence studies among animals (10309 animals), and 9 studies on vectors (184242 vector species). Of the 22 countries in this region, five had no data on WNV infection among different populations. These countries include Kuwait, Bahrain, Oman, Syria and Somalia. On the other hand, among countries with available data, WNV-specific antibodies were detected in the general population of all investigated countries including Djibouti (0.3–60%), Egypt (1–61%), Iran (0–30%), Iraq (11.6–15.1%), Jordan (8%), Lebanon (0.5–1%), Libya (2.3%), Morocco (0–18.8%), Pakistan (0.6–65.0%), Sudan (2.2–47%), and Tunisia (4.3–31.1%). WNV RNA were also detected in patient populations of Iran (1.2%), Pakistan (33.3%), and Tunisia (5.3% –15.9%). WNV-specific antibodies were also detected in a wide range of animal species. The highest seropositivity rate was observed among equids (100% in Morocco) and dogs (96% in Morocco). The highest seroprevalence among birds was seen in Tunisia (23%). In addition, WNV infection was detected in mosquitoes (*Culex*, and *Aedes*) and ticks (*Argas reflexus hermanni*). The primary vector of WNV (*Culex pipiens* s.l.) was detected in Djibouti, Egypt, Iran and Tunisia, and in mosquitoes of all these countries, WNV was demonstrated.

**Conclusions:**

This first systematic regional assessment of WNV prevalence provides evidence to support the circulation of WNV in the EMRO region as nearly all studies showed evidence of WNV infection in human as well as animal/vector populations. These findings highlight the need for continued prevention and control strategies and the collection of epidemiologic data for WNV epidemic status, especially in countries that lack reliable surveillance systems.

## Author summary

West Nile Virus (WNV) is a mosquito-borne *Flavivirus* belonging to the *Flaviviridae* family, which is endemic in a vast geographical area, including the EMRO region. However, the epidemiology of WNV in the EMRO region remains poorly understood. To address this gap, we performed a systematic review on WNV prevalence studies conducted on human populations, animals and vectors across Eastern Mediterranean countries. Our review indicated the infection of most investigated human, animal and vector populations with WNV; however, the paucity of epidemiological data underline the need for integrated surveillance programs as well as continued deployment of prevention and control strategies.

## Introduction

West Nile Virus (WNV) is one of the most widely distributed arboviruses in the world, and a pathogen of public health significance in both humans and animals [1]. This mosquito-borne virus has been classified in the genus *Flavivirus* within the family *Flaviviridae* [2]. In nature, WNV is maintained in a zoonotic transmission cycle between birds and mosquitos, principally the *Culex* species. Susceptibility to WNV infection has also been indicated for many other vertebrate hosts including mammals, birds, reptiles, and amphibians [3]. Equines and humans are incidental “dead-end” hosts who do not play a role in the transmission cycle of the virus. However, equines and humans may manifest sever disease or death as a consequence of infection [4]. Since the first discovery of the virus in 1937 in the West Nile district of Uganda [5], it has undergone a substantial geographical migration, and spread around the globe. Infection with WNV was first identified in an EMRO country (Sudan) in the 1940s. Since then, infection with the virus has been reported in Egypt (1950s), Iran (1970s), and subsequently in several other countries across the region [6]. The prevention and control efforts substantially rely on effective surveillance of the infection in birds, vectors, animals, and humans. Despite several studies on different aspects of WNV epidemiology in the EMRO region, there are still many unknowns about the circulation of the virus and the driving factors of outbreaks [6, 7]. Understanding the epidemiology of WNV in the EMRO faces a number of challenges including inadequate knowledge of physicians about the nature of the disease, misdiagnosis of other common infectious diseases due to similarity in clinical presentations, poor diagnostic infrastructures and the absence of confirmatory assays for serological tests, and lack of a comprehensive and progressive monitoring and surveillance system in majority of countries. The latter has resulted in a gap in knowledge regading the prevalence of WNV infection in the EMRO region. Therefore, we designed a systematic review to provide a clear and comprehensive presentation of the virus prevalence distribution among human and animal populations as well as infection rate in vectors of the region, based on available data.

## Methods

### Data sources and search strategy

Articles were screened and selected according to the PRISMA criteria [8]. The PRISMA checklist completed for this review is presented in S1 File. We made an electronic literature search through Web of Science, Scopus, PubMed, Google Scholar, and Index Medicus for the Eastern Mediterranean region database (IMEMR) using different combinations of the following keywords ‘West Nile virus, West Nile Fever, WNV’ and the name of the EMRO countries as: Afghanistan, Bahrain, Djibouti, Egypt, Iran, Iraq, Jordan, Kuwait, Lebanon, Libya, Morocco, Oman, Pakistan, Palestine, Qatar, Saudi Arabia, Somalia, Sudan, Syria, Tunisia, United Arab Emirates, and Yemen (S2 File). All databases were searched for English-language original articles published from database inception to January 30, 2018. Choosing multiple sources for article search we aimed to enhance our sensitivity in finding relevant articles. To find citations that were not indexed in our target databases, we reviewed the reference lists of relevant articles.

### Review selection

Studies identified through electronic and manual searches were listed in EndNote software (EndNote X7, Thomson Reuters). After exclusion of duplicate citations, two authors (MF, FS) independently reviewed titles and abstracts according to the research question. Relevant studies were obtained in full, and assessed for eligibility and risk of bias as described below. All original articles from peer-reviewed scientific journals with a cross-sectional or survey design that estimated the prevalence of WNV infection in humans, animals, or infection rate in vectors were potentially eligible for inclusion in this review. Relevant studies whose abstract was available but their full-text was not (even after contacting the authors via e-mail), were kept in this review in order to present all available data. Studies from outside of the EMRO region were excluded. Any disagreements between the review team were resolved through discussion.

### Risk of bias assessment

The risk of bias in primary studies was assessed following the Cochrane approach [9]. We also considered individual studies’ sample size (precision) as a criterion to assess risk of bias, as proposed by Humphre, et al. [10]. Therefore, we evaluated each WNV prevalence study in three domains: 1) sampling method, 2) response level (the proportion of subjects who accept to participate in the study), and 3) type of assay used for the detection of WNV. Each study was considered to have a low risk of bias if: 1) it used probability-base/random sampling methods, 2) maintained participants’ response level at ≥80% [11, 12], or 3) it employed viral neutralization testing (VNT) for a prevalence study on the general population or used biological tests including viral genome detection and virus isolation from infected individuals. Studies were classified as having unclear risk of bias for a given domain if they did not provide information for that specific domain. Use of probabilistic sampling methods was only evaluated for studies on the general population, because acute infection studies included individuals attending to healthcare facilities. For studies that were conducted on blood samples collected and stored from blood donors, response rate criteria were not evaluated. Studies on human subjects were considered to have high precision if their sample sizes were ≥ 100 [13]. Moreover, in the studies on WNV vectors, minimum infection rates (MIR), that were calculated for samples of ≥ 1000 specimens, were considered as a reliable representation of the true infection rate in the vector population [14, 15].

### Data extraction

Data was extracted from the selected studies using a researcher-made and piloted data extraction form in excel. For studies on human and animal subjects we extracted data on: first author, year of publication, year of implementation, country, city/governorate, sample size, participants’ age and sex (for human subjects only), animal species (for studies on animals), assay type, and estimated assay-based WNV prevalence. For studies on vector populations, further data was extracted including vector species, number of species (vectors) tested, collection methods, number and size of the pools as well as the number of positive pools for each species. WNV minimum infection rate (MIR) for each species was calculated by dividing the number of positive pools by the total number of specimens tested for that specific species and multiplied by 1000. When data was available, assay-specific MIRs were calculated and reported.

## Results

### Search results

**Fig 1:**
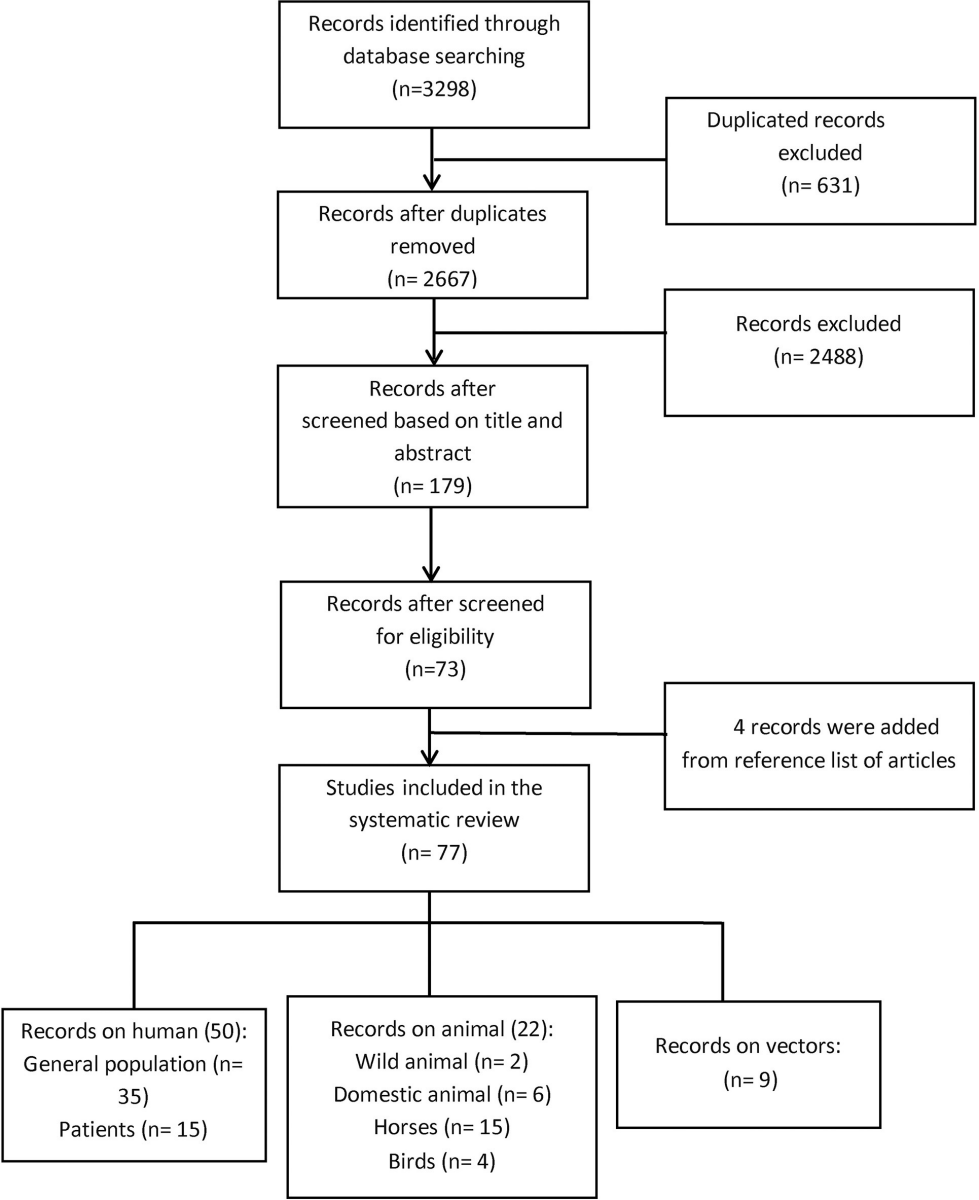
Flow diagram of article selection for West Nile prevalence in human and animals, and infection rate in vectors of the EMRO region. Four citations included data on more than one subject categories (*i*.*e*., humans, animal species, and vectors).

Database search resulted in 3298 records. After removal of duplicates, we initially screened the title and abstract of 2667 records, 2488 of which were excluded as they were irrelevant to this review. The remaining 179 papers were reviewed in full, of which 77 eligible reports on the prevalence/MIR of WNV covering 17 countries in the EMRO region were included in this systematic review. We identified two relevant citations by reviewing the reference list of these relevant studies [16, 17]. Fig 1 shows the literature search process. The full-text of five studies could not be obtained even after contacting the authors [18–22]. These studies were kept in this review to present all available data to the readers. All included studies on WNV entailed 27899 individuals (24460 general populations and 3439 patients), 10309 animals, and 184242 vector species.

### Risk of bias assessment results

A summary of the risk of bias assessment results is shown in Table 1. In brief, most human studies (28 out of 35) contained sample sizes of ≥100 participants, yielding a high precision in the reported prevalence measure. Thirty out of thirty-five studies on the general population reported their sampling strategy, fourteen of which utilized some forms of random sampling, and hence, had low risk of bias at this domain. In most studies on the general population (24 out of 35), risk of bias assessment was affected by unclear reporting in the ‘response rate’ domain. Six studies were performed on volunteers or on blood specimens stored in national reference laboratories or blood transfusion centers, and hence, were not subjected to risk of bias assessment in the ‘response rate’ domain. Viral neutralization test was performed in 40.0% and 13.3% of prevalence studies on the general and patient populations, respectively, which entails a low risk of bias for the assays used.

**Table 1:** Precision and risk of bias assessment for West Nile virus prevalence measures in the EMRO region.

Author, Year	Country	Sampling Method^¥^	Risk of bias			Precision	Ref.
			In sampling^¥^	In response rate	In assay selection		
**General population**							
**Andayi, 2014**	Djibouti	Random	Low	Unclear	Low	High	[23]
**Faulde, 2012**	Djibouti	Conv.	Low	Low (100%)	High	Low	[24]
**Youssef, 2017**	Egypt	Conv.	High	Unclear	High	High	[25]
**Soliman, 2010**	Egypt	Random	Low	Unclear	Low	High	[26]
**Darwish 1996**	Egypt	Unclear	Unclear	Unclear	High	High	[22]
**Corwin, 1993**	Egypt	CS	Low	Low (93%)	High	High	[27]
**Corwin, 1992**	Egypt	Random	Low	Low (78%)	High	High	[28]
**Darwish, 1975**	Egypt	CS	Low	Unclear	High	High	[29]
**Taylor, 1956**	Egypt	CS	Low	Unclear	Low	High	[30]
**Aghaie, 2016**	Iran	Conv.	High	Unclear	High	High	[31]
**Meshkat, 2015**	Iran	MSCS	Low	Unclear	High	High	[32]
**Chinikar, 2013**	Iran	Unclear	Unclear	Unclear	High	Low	[33]
**Sharifi, 2010**	Iran	Conv.	High	Low (100%)*	High	High	[34]
**Saidi, 1976**	Iran	Random	Low	Unclear	Low	High	[17]
**Saidi,1974**	Iran	Random	Low	Unclear	NA	High	[16]
**Naficy, 1970**	Iran	Unclear	Unclear	Unclear	Low	High	[19]
**Barakat, 2016**	Iraq	Conv.	High	Low (100%)**	Low	High	[35]
**Batieha, 2000**	Jordan	Conv.	High	56%	High	High	[36]
**Gallian, 2010**	Lebanon	Conv.	High	Unclear	Low	High	[37]
**Garabedian, 1971**^**ǂ**^	Lebanon	Unclear	Unclear	Unclear	High	High	[18]
**Shaibi, 2017**	Libya	Random	Low	Unclear	High	High	[38]
**El Harrak 2016**^**ǂ**^	Morocco	Conv.	High	Unclear	Low	High	[39]
**El Rhaffouli, 2013**	Morocco	Conv.	High	Low (100%)*	Low	High	[40]
**El Rhaffouli, 2012**	Morocco	Random	Low	Low (100%)	Low	High	[41]
**Niazi 2017**	Pakistan	Random	Low	Unclear	High	High	[42]
**Sugamata, 1989**	Pakistan	Unclear	Unclear	Unclear	Low	High	[43]
**Sugamata, 1988**	Pakistan	Unclear	Unclear	Unclear	Low	Low	[44]
**Darwish, 1983**	Pakistan	Conv.	High	Unclear	High	Low	[45]
**Hayes, 1982**	Pakistan	Conv.	High	Low (100%)**	Low	High	[46]
**Yousof 2017**	Sudan	Random	Low	Low (100%)*	High	Low	[47]
**Farnon 2010**	Sudan	Conv.	High	Unclear	Low	Low	[48]
**Salim, 1973**	Sudan	Conv.	High	Unclear	Low	High	[49]
**Taylor, 1956**	Egypt	CS	Low	Unclear	Low	High	[30]
**Smithbur, 1942**	Anglo- Egyptian Sudan	CS	Low	Unclear	Low	High	[50]
**Riabi, 2010**	Tunisia	Conv.	High	Low (100%)**	Low	High	[51]
**Alfaresi, 2008**	UAE	Conv.	High	Unclear	High	Low	[52]
**Patients**							
**Elyan, 2014**	Afghanistan	NA	NA	Unclear	High	High	[53]
**Darwish, 1987**	Egypt	NA	NA	Unclear	High	Low	[54]
**Mohammed, 1970**	Egypt	NA	NA	Low (100%)	High	High	[55]
**Abdel Wahab, 1970**^**ǂ**^	Egypt	NA	NA	Unclear	NA	High	[20]
**Chinikar, 2012**	Iran	NA	NA	Unclear	Low	High	[56]
**Yaqub,2017**	Pakistan	NA	NA	Low (100%)^€^	Low	High	[57]
**Khan, 2016**	Pakistan	NA	NA	100%	High	High	[58]
**Bryan, 1996**^**ǂ**^	Pakistan	NA	NA	Unclear	NA	High	[21]
**Igarashi, 1994**	Pakistan	NA	NA	Unclear	Low	High	[59]
**Depoortere, 2004**	Sudan	NA	NA	Low (100%)^€^	High	Low	[60]
**McCarthy, 1996**	Sudan	NA	NA	Unclear	High	High	[61]
**Watts, 1994**	Sudan	NA	NA	Unclear	High	High	[62]
**Riabi, 2014**	Tunisia	NA	NA	Unclear	Low	High	[63]
**Feki, 2005**	Tunisia	NA	NA	Low (100%)	Low	Low	[64]
**Qassem, 2014**	Yemen	NA	NA	Unclear	High	Low	[65]

* On blood specimens stored in the blood transfusion center

** On volunteers

^ǂ^ Studies were classified as having “Unclear” risk of bias for a given domain if they did not provide information for that specific domain. These studies were categorized as “Unclear” risk of bias.

^¥^ Use of probabilistic sampling methods was only evaluated for studies on the general population, because acute infection studies included individuals attending to healthcare facilities. So, risk of bias assessment for the “sampling” domain, was “Not Applicable” (NA) for patients.

^€^ On archived samples.

Abbreviations: **Conv**: Convenience sampling. **CS**: Cluster Sampling. **MSCS**: Multi-stage cluster sampling. **NA**: Not applicable to the field.

### WNV prevalence among the general and patient populations of the EMRO region

**Fig 2:**
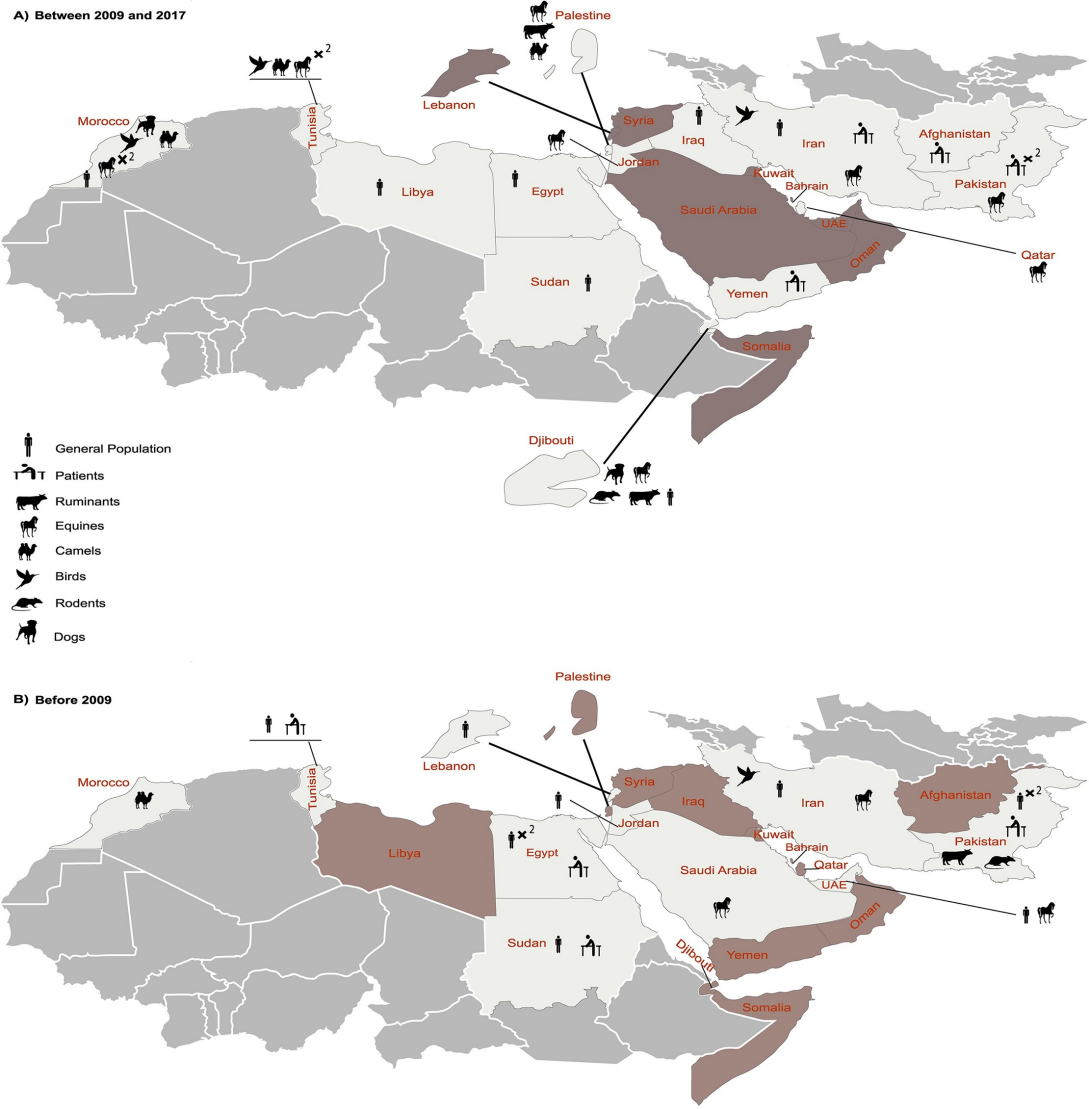
WNV infection reported among human and animal populations of the EMRO countries. A) studies conducted between 2009 and 2017; B) studies conducted before 2009. Black symbols indicate samples with evidence of WNV infection. The exact sampling location and the prevalence values are provided in the Tables 2 and 3. Countries with no qualified study on human or animal populations are colored in gray. ×2: Where more than one study/sampling effort have been done on a particular species in the same geographic area. UAE: United Arab Emirates.

A total of 50 human prevalence studies for WNV were identified, 35 of which estimated the seroprevalence in the general population. Furthermore, and 15 of them investigated the presence of WNV antibody or genetic material in patients suspected with WNV infection. Human studies covered 14 of 22 countries of the EMRO region, and were published from 1942 to 2017. The highest number of human studies were reported from Egypt (n = 10), Iran (n = 8), and Pakistan (n = 9), most of which targeted the general population. ELISAs were the most commonly used diagnostic method for the general and patient populations. Table 2 presents detailed data for these studies. The geographic distribution of human prevalence studies is also illustrated in Fig 2A and 2B.

**Table 2:** Summary of human prevalence studies for West Nile virus in the EMRO region (n = 50).

Author, Pub. year	Study year	Country	City/governorate		SS	Participant characteristics		Assay	Prevalence (%)							Ref
						Male/Female	Age range (yrs)		IgG ELISA	IgM ELISA	NT	IF	HI	CF	RT-PCR	
**General population**																
**Andayi, 2014**	2010–2011	Djibouti	Djibouti		893^§^	NA	NA	ELISA, NT	0.6	-	0.3	-	-	-	-	[23]
**Faulde, 2012**	2010–2012	Djibouti	Djibouti		10	NA	NA	IF	-	-	-	60.0	-	-	-	[24]
**Youssef, 2017**	2013–2014	Egypt	NA		160	124/ 36	18–55	ELISA, RT-PCR	55.0	-	-	-	-	-	0	[25]
**Soliman, 2010**	1999–2000	Egypt	Total		5965	NA	NA	ELISA, NT	24.0	-	24.0	-	-	-	-	[26]
			Fayoum		1593				27.3	-	27.3	-	-	-	-	
			Sharqiya		1292				13.8	-	13.8	-	-	-	-	
			Al Arish		202				1.0	-	1.0	-	-	-	-	
			Nuweiba		675				6.7	-	6.7	-	-	-	-	
			Qena		2203				35.0	-	35.0	-	-	-	-	
**Darwish, 1996**^**ǂ**^	NA	Egypt	Minufiya		178	NA	NA	ELISA, HI, IF	45.0	-	-	26.4	37.6	-	-	[22]
**Corwin, 1993**	1991	Egypt	Nile		915	356/559	<1–80	ELISA	20.0	-	-	-	-	-	-	[27]
**Corwin, 1992**	1989	Egypt	Nile		437	215/222	8–14	ELISA	3.0	-	-	-	-	-	-	[28]
**Darwish, 1975**	1969	Egypt	Cairo		1133	NA	NA	HI	-	-	-	-	50.0	-	-	[29]
**Taylor, 1956**	NA	Egypt	Egyptian Nile delta		1168	NA	NA	NT	61	-	61	-	-	-	-	[30]
**Aghaie, 2016**	NA	Iran	Chabahar		540	514/26	17–65	ELISA,IF	18.0	-	-	1.5	-	-	-	[31]
**Meshkat, 2015**	2011–2012	Iran	Mashhad		182	46/136	15–65	ELISA	11.0	-	-	-	-	-	-	[32]
**Chinikar, 2013**	2010–2011	Iran	Total		300	NA	NA	ELISA	1.3	-	-	-	-	-	-	[33]
			Golestan		90				2.2	-	-	-	-	-	-	
			Gilan		70				1.4	-	-	-	-	-	-	
			Mazandaran		71				0.0	-	-	-	-	-	-	
			Qom		69				1.4	-	-	-	-	-	-	
**Sharifi, 2010**	2005	Iran	Tehran		500	490/10	17–65	ELISA, RT-PCR	5.0	0	-	-	-	-	0	[34]
**Saidi, 1976**	1971–1975	Iran	NA		698	NA	NA	NT	-	-	26.6	-	-	-	-	[17]
**Saidi,1974**	NA	Iran	NA		100	NA	NA	NA	10.0	-	-	-	-	-	-	[16]
**Naficy, 1970**^**ǂ**^	NA	Iran	NA		2975	NA	NA	NT, HI	30.0	-	-	-	-	-	-	[19]
**Barakat, 2016**	2012–2013	Iraq	Nasiriyah		397	NA	10–82	NT, IF, HI	-	-	11.6	14.9	15.1	-	-	[35]
**Batieha, 2000**	1998	Jordan	Hashimia		261	75/186	≥ 5	ELISA	8.0	-	-	-	-	-	-	[36]
**Gallian, 2010**	2006	Lebanon	Total		627	74/553	18–59	ELISA,NT	1.0	-	0.5	-	-	-	-	[37]
			Central Lebanon		500											
			Bekaa		36											
			Northern Lebanon		46											
			Southern Lebanon		45											
**Garabedian, 1971**^**ǂ**^	NA	Lebanon	NA		215	NA	NA	HA, CF	NA	-	-	-	0	0	-	[18]
**Shaibi, 2017**	2013	Libya	Tripoli		400	277/123	15–78	ELISA	2.3	-	-	-	-	-	-	[38]
**El Harrak, 2016**	2013	Morocco	NA		622	NA	NA	ELISA, NT	-	0	5.6	-	-	-	-	[39]
**El Rhaffouli, 2013**	2012	Morocco	Wad-ad-Dahab		250	147/103	<1–80	NT	-	-	5.2	-	-	-	-	[40]
**El Rhaffouli, 2012**	2011	Morocco	Total		499	186/313	31–65	NT	-	-	11.8	-	-	-	-	[41]
			Meknes		150	46/104	37–67		-	-	4.7	-	-	-	-	
			Rabat		200	76/124	37–61		-	-	12.0	-	-	-	-	
			Kenitra		149	64/85	31–65		-	-	18.8	-	-	-	-	
**Niazi, 2017**	NA	Pakistan	NA		1860	1847/13	18–57	RT-PCR	-	-	-	-	-	-	0.21	[42]
**Sugamata, 1989**	1985	Pakistan	Karachi		150	NA	6–65	NT	-	-	53.3	-	-	-	-	[43]
**Sugamata, 1988**	1983	Pakistan	Karachi	July	33	14/19	NA	HI	-	-	-	-	55.0	-	-	[44]
				September	48	29/15	NA	HI	-	-	-	-	65.0	-	-	
	1985		Karachi	July	156	122/34	NA	NT, HI	-	-	50.0	-	53.0	-	-	
				October	156	122/34	NA	NT, HI	-	-	54.0	-	59.0	-	-	
**Darwish, 1983**	NA	Pakistan	Karachi, Sind, Punjab		43	NA	NA	CF	-	-	-	-	-	11.6	-	[45]
**Hayes, 1982**	1978–1979	Pakistan	Chiniot		192	NA	1->61	NT	-	-	32.8	-	-	-	-	[46]
			Change Manga national forest		239	NA	1->61	NT, HI	-	-	38.5	-	33.1	-	-	
**Yousof, 2017**	2016	Sudan	Khartoum		90	NA	NA	ELISA	44.4	2.2	-	-	-	-	-	[47]
**Farnon, 2010**	2005	Sudan	Kortalla		87	37/50	5–44<	NT	-	-	39.1	-	-	-	-	[48]
**Salim, 1973**	NA	Sudan	Sennar		17	NA	<1–40	NT	-	-	47.0	-	-	-	-	[49]
**Taylor, 1956**	NA	Sudan	Southern Sudan		350	NA	NA	NT	40	-	40	-	-	-	-	[30]
**Smithburn, 1942**	1939–1940	Anglo-Egyptian Sudan	Total		270	NA	4–75	NT	-	-	28.9	-	-	-	-	[50]
			Red Sea coast		23		5–55		-	-	13.0	-	-	-	-	
			Eastern border		75		4–60		-	-	33.3	-	-	-	-	
			White Nile		56		6–75		-	-	46.4	-	-	-	-	
			Kordofan		89		4–68		-	-	20.8	-	-	-	-	
			Southwestern		27		7–40		-	-	18.6	-	-	-	-	
**Riabi, 2010**	2003	Tunisia	Monastir		742	497/245	18–54	ELISA,NT	15.6	-	4.3	-	-	-	-	[51]
			Mahdia		102	68/34	19–45		31.1	-	13.7	-	-	-	-	
**Alfaresi, 2008**	2005	UAE	UAE		500	-	-	RT-PCR	-	-	-	-	-	-	0	[52]
**Patients**																
**Elyan, 2014**	2008–2010	Afghanistan	Uruzgon, Helmand, Kandahar, Kabul		913	493/420	20–59	ELISA, NT	30.4	0.5	2.6	-	-	-	-	[53]
**Mohammed, 1970**	1968	Egypt	Alexandria	Acute sample	120	60/60	3–13	CF, HI	-	-	-	-	4.3	0	-	[55]
				Convalescent sample	48	24/24	NA	HI	-	-	-	-	14.6	-	-	
**Darwish, 1987**	1985	Egypt	Cairo	Prior infection	55	32/23	>10	HI	-	-	-	-	58.0	-	-	[54]
				Acute infection					-	-	-	-	1.8	-	-	
**Abdel Wahab,1970**^**ǂ**^	NA	Egypt	NA		133	NA	NA	NA	3.7	-	-	-	-	-	-	[20]
**Chinikar, 2012**	2008–2009	Iran	Isfahan		249	126/123	10–81	ELISA, RT-PCR	2.4	0	-	-	-	-	1.2	[56]
**Yaquba, 2017**	2014–2015	Pakistan	Rawalpindi/Islamabad, Lahore, and Faisalabad		480	NA	NA	ELISA, RT-PCR	1.3	-	-	-	-	3.1	-	[57]
**Khan, 2016**	2015	Pakistan	Karachi, Hyderabad, Mirpurkhas, Sukkur		241	NA	10–50	ELISA	-	6.6	-	-	-	-	-	[58]
**Bryan, 1996**^**ǂ**^	NA	Pakistan	NA		570	NA	NA	NA	33–41	-	-	-	-	-	-	[21]
**Igarashi, 1994**	1992	Pakistan	Karachi		24	NA	NA	ELISA,RT-PCR	-	0	-	-	-	-	33.3	[59]
**Depoortere, 2004**	2002	Sudan	Ngorban, South Kordophan	Neurological sequelae	8	7/6	6–84	ELISA, NT	62.5	87.5	6.6	-	-	-	-	[60]
				Convalescent	5				0	20.0	0.1	-	-	-	-	
**McCarthy, 1996**	1988	Sudan	Khartoum		196	NA	1–89	ELISA	60.0	0.3	-	-	-	-	-	[61]
**Watts, 1994**	1989	Sudan	Karima		185	NA	11–70	ELISA	59.0	-	-	-	-	-	-	[62]
**Riabi, 2014**	2003	Tunisia	Monastir		113	NA	NA	ELISA, RT-PCR	33.6		-	-	-	-	15.9	[63]
**Feki, 2005**	1997	Tunisia	Sfax		57	50/7	NA	ELISA, RT-PCR	52.6		-	-	-	-	5.3	[64]
**Qassem, 2014**	2013	Yemen	NA		42	NA	All	ELISA	-	14.3	-	-	-	-	-	[65]

^**§**^ These individuals are selected from 324 households.

^**ǂ**^ The data for these studies is driven from articles’ abstract, as their full-text could not be obtained.

Abbreviations: **SS:** Sample Size, **ELISA:** Enzyme-linked Immunosorbent Assay, **NT:** Neutralization Test, **IF:** Immunofluorescence Assay, **HI:** Hemagglutination Inhibition Assay, **CF:** Complement Fixation, **RT-PCR:** Reverse Transcriptase-Polymerase Chain Reaction, **NA:** Data was not available, **UAE:** United Arab Emirates.

Regarding the general population, WNV antibodies were detected in 11 countries including Djibouti (n = 2, 0.3–60%), Egypt (n = 7, 1–61%), Iran (n = 6, 0–30%), Iraq (n = 1, 11.6–15.1%), Jordan (n = 1, 8%), Lebanon (n = 2, 0–1%), Libya (n = 1, 2.3%), Morocco (n = 3, 0–18.8%), Pakistan (n = 5, 0.2–65%), Sudan (n = 5, 2.2–47%), and Tunisia (n = 1, 4.3–31.1%). Since 2010, seroprevalence of WNV among the general population has been investigated in Djibouti, Egypt, Iran, Iraq, Libya, Morocco, and Sudan among which the lowest and highest median prevalence was found in Iran (median prevalence = 1.4, range: 0–18%; total SS = 1322; 2010–2012), and Egypt (median prevalence = 55%; total SS = 160, 2013–2014), respectively (Table 2).

In addition, the presence of WNV antibody or genetic material in patients was investigated in 15 human prevalence studies. In this regard, seven studies assessed WNV IgM, five of which detected the antibodies in patients’ sera. These studies were from Afghanistan (n = 1, 0.5%), Pakistan (n = 1, 6.6%), Sudan (n = 2, 0.3–87.5%), and Yemen (n = 1, 14.3%). Four studies [56, 59, 63, 64] used both serological and molecular assays to detect WNV IgM as well as WNV RNA in patients’ sera (Table 2).

### WNV prevalence in wild and domestic animals in the EMRO region

A total of 22 studies investigated the WNV seroprevalence in animals (Fig 2A and 2B). WNV antibodies were detected in 10 countries, including, Djibouti (n = 1), Iran (n = 4), Jordan (n = 1), Morocco (n = 4), Pakistan (n = 2), Palestine (n = 1), Qatar (n = 2), Saudi Arabia (n = 1), Tunisia (n = 5) and the United Arab Emirates (UAE; n = 1). In these studies, serological evidence of WNV infection was detected in a wide range of domestic and wild animals, including Buffalos (Pakistan, total SS = 33, prevalence = 15.1%), Camels (Morocco, total SS = 2775, prevalence = 8–23%; Palestine, total SS = 35, Prevalence = 40%; Tunisia, total SS = 120, Prevalence = 0–25.8%), Cows (Pakistan, total SS = 45, prevalence = 2.2%), Goats and sheep (Pakistan, total SS = 94, prevalence = 23.9%; Palestine, total SS = 95, prevalence = 14.7%), Dogs (Djibouti, total SS = 91, prevalence = 56.5%; Morocco, total SS = 231, prevalence = 54–96%), Ruminants (Djibouti, total SS = 11, prevalence = 25.3%), Equids (Iran, total SS = 1839, prevalence = 0.8–70.3%; Jordan, total SS = 253, prevalence = 24.9%; Morocco, total SS = 1189, prevalence = 25–100%; Pakistan, total SS = 449, prevalence = 65%; Palestine, total SS = 585, prevalence = 75%; Qatar, total SS = 421, prevalence: 0–27%; Saudi Arabia, total SS = 63, prevalence = 33.5%; Tunisia, total SS = 1473, prevalence = 28.0–45.2%; the UAE, total SS = 750, prevalence = 5.4–28.6%), and different types of wild and domestic birds (Iran, total SS = 519, prevalence = 15%; Morocco, total SS = 346, prevalence = 3.5%; Tunisia, total SS = 434, prevalence = 0.7–23%). Table 3 provides further details on these studies.

**Table 3:** Summary of animal prevalence studies for West Nile virus in the EMRO region (n = 22).

Author, Pub. year		Study year	Country	City/governorate	Species	SS	Prevalence (%)							Ref
							ELISA			NT	IF	HI	CF	
							IgM	IgG	cELISA					
**Domestic animals**														
**Marié, 2016**	2012–2016		Djibouti	Djibouti	Dog, Ruminant	91	-	56.5	-	-	-	-	-	[66]
**Durand, 2016**	2012		Morocco	Total	Dog	231	-	-	62.0	80.0 *	-	-	-	[67]
				Benslimane			-	-	54.0		-	-	-	
				Kenitra			-	-	96.0		-	-	-	
				Khenifra			-	-	75.0		-	-	-	
				Sidi Slimane			-	-	94.5		-	-	-	
**Touil, 2012**	2003		Morocco	Total	Camel	556	-	-	-	10.4	-	-	-	[68]
				Guelmim (Atlantic littoral)		73	-	-	-	23.0	-	-	-	
				Smara (Sahara)		85	-	-	-	8.0	-	-	-	
				Aousserd (Sahara)		389	-	-	-	9.0	-	-	-	
	2009			Total		836	-	-	-	13.6	-	-	-	
				Oued Draa		157	-	-	-	12.0	-	-	-	
				Laayoune (Atlantic littoral)		397	-	-	-	15.0	-	-	-	
				Smara (Sahara)		282	-	-	-	12.0	-	-	-	
**Darwish, 1983**	1983		Pakistan	Karachi, Sind, Punjab	Total	172	-	-	-	-	-	-	9.9	[45]
					Cow	45	-	-	-	-	-	-	2.2	
					Buffalo	33	-	-	-	-	-	-	15.1	
					Sheep	46	-	-	-	-	-	-	23.9	
					Goat	48	-	-	-	-	-	-	0	
**Azmi, 2017**	2014		Palestine	Nablus, Jericho, and Jenin	Goat, Sheep	95	-	-	14.7	-	-	-	-	[69]
					Camel	35	-	-	40.0	-	-	-	-	
**Hassine, 2017**	2016		Tunisia	Medenine	Camel	87	-	-	0.0	-	-	-	-	[70]
				Kebili		31	-	-	25.8	-	-	-	-	
**Wild animals**														
**Marié, 2016**	2016		Djibouti	Djibouti	Ruminants	11	-	25.3	-	-	-	-	-	[66]
**Darwish, 1983**	1983		Pakistan	Karachi	Rodents	157	-	-	-	-	-	-	4.5	[45]
**Horses**														
**Marié, 2016**	2016		Djibouti	Djibouti	Equids	10	-	90.0	-	-	-	-	-	[66]
**Pourmahdi, 2013**	2011–2012		Iran	Khuzestan Province	Horse	155	-	-	70.3	-	-	-	-	[71]
**Chinikar, 2013**	2010–2012		Iran	Total	Equine	315	-	2.8	-	-	-	-	-	[33]
				Golestan		65	-	6.1	-	-	-	-	-	
				Gilan		98	-	2.0	-	-	-	-	-	
				Isfahan		152	-	1.9	-	-	-	-	-	
**Ahmadnejad, 2011**	2008–2009		Iran	27 provinces of Iran	Equine	1054	0.8	-	-	23.6	-	-	-	[72]
**Abutarbush, 2014**	2012		Jordan	Irbid, Ajlun and Jerash	Horse	253	-	-	-	24.9	0	-	-	[73]
				Amman and Madaba										
				Ma’an, Karak, Tafelah and Aqaba										
				Mafraq and Zarka										
				Jordan Valley and Balqa										
**Benjelloun, 2017**	2011		Morocco	4 zones	Horse	840	-	-	31.0	-	-	-	-	[74]
**Durand, 2016**	2012		Morocco	Total	Horse	349	-	60.0	-	74.0	-	-	-	[67]
				Agadir			-	65.0	-		-	-	-	
				Benslimane			-	25.0	-		-	-	-	
				Casablanca			-	75.0	-		-	-	-	
				Kenitra			-	82.0	-		-	-	-	
				Khenifra			-	29.0	-		-	-	-	
				Marrakech			-	32.0	-		-	-	-	
				Meknes			-	34.0	-		-	-	-	
				Salé			-	50.0	-		-	-	-	
				Sidi Slimane			-	100	-		-	-	-	
				Temara			-	94.0	-		-	-	-	
**Zohaib, 2015**	2012–2013		Pakistan	Punjab, Khyber Pakhtunkhwa	Equine	449	-	65·0	-	55·4	-	-	-	[75]
**Azmi, 2017**	2014		Palestine	NA	Equids	585	-	-	75.0	-	-	-	-	[69]
**DeCarlo, 2017**	NA		Qatar	Throughout the country	Horse	161	-	-	27.0	-	-	-	-	[76]
**Haroun, 2017**	2006–2014		Qatar	Qatar	Horse	260	0	-	23.5	-	-	-	-	[77]
**Al-Ghamdi, 2014**	2007		Saudi Arabia	Al-Ahsa	Horse	63	-	33.3		-	-	-	-	[78]
**Bargaoui, 2015**	2009		Tunisia	Jendouba, Monastir, Chott El Jerid, Chott el Gharsa	Equine	1189	-	28.0	-	-	-	-	-	[79]
**Ben Hassine, 2014**	2012		Tunisia	Kebili	Equine	284	-	45.2	-	42.3	-	-	-	[80]
**Wernery, 2007**	NA		UAE	Total	Equine	750	-	-	19.2	-	-	-	-	[81]
				Al Fujairah			-		11.5					
				Ras Al Khaimah			-		5.4					
				Ajman			-		7.1					
				Sharjah			-		8.2					
				Dubai			-		10.0					
				Al Ain			-		12.0					
				Abu Dhabi			-		28.6					
**Birds**														
**Fereidouni, 2011**	2003–2007		Iran	Mazandaran, Gilan, West Azerbaijan, Tehran, Fars, Khuzestan	27 species	NT+IF = 519; RT-PCR = 400	-	-	-	15.0		-	0	[82]
**Figuerola, 2009**	2008		Morocco	Sidi Allal Tazi, Sidi Kacem	Wild birds	346	-	-	-	3.5	-	-	-	[83]
**Hammouda, 2015**	2012–2015		Tunisia	Gabès	Wild Sparrow	154	-	0.7	-	0.7	-	-	-	
				Kébili oases		54	-	1.9	-	1.9	-	-	-	[84]
**Ayadi, 2017**	2015		Tunisia	Total	Laughing doves	226	-	-	17.0	10	-	-	-	[85]
				Kettana		102	-	-	23.0	15	-	-	-	
				Gafsa		53	-	-	13.0	6.0	-	-	-	
				Degache		26	-	-	4.0	4.0	-	-	-	
				Oum-Errous		45	-	-	16.0	7.0	-	-	-	

* 80% of ELISA positive samples were positive by NT.

Abbreviations: **SS:** Sample Size, **ELISA:** Enzyme-linked Immunosorbent Assay, **cELISA:** Competitive ELISA, **NT:** Neutralization Test, **IF:** Immunofluorescence Assay, **HI:** Hemagglutination Inhibition Assay, **CF:** Complement Fixation, **RT-PCR:** Reverse Transcriptase-Polymerase Chain Reaction, **NA:** Data was not available, **UAE:** United Arab Emirates.

### Infection rate of vectors with WNV in the EMRO region

**Fig 3:**
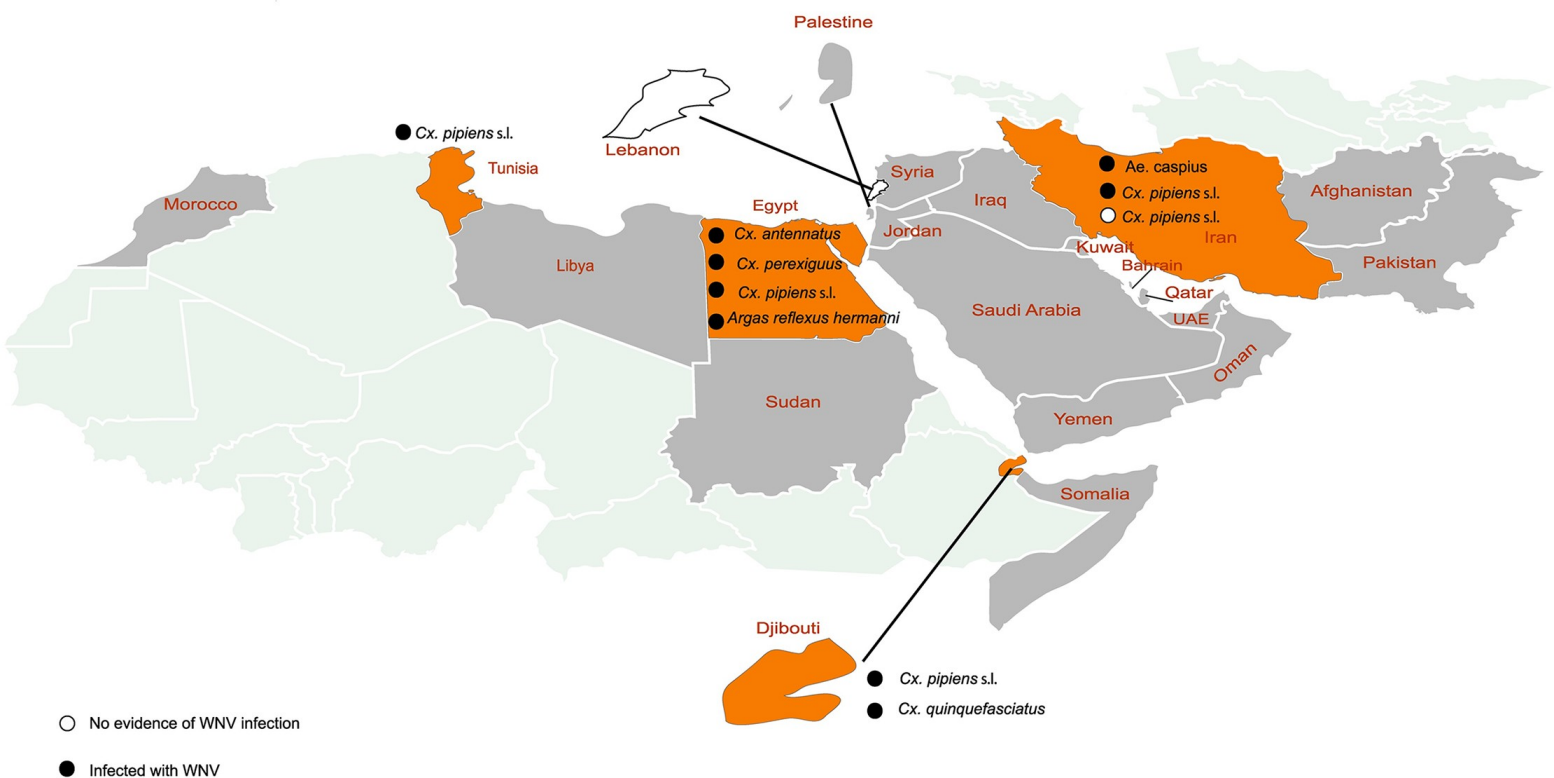
Vector infection with the WNV in the EMRO region. From each study, only the names of vector species with evidence of WNV infection are written on the map. Countries, from which the main vector for WNV (*i*.*e*., *Cx*. *pipiens* s.l.) was detected, are colored in orange. These countries include Djibouti, Egypt, Iran, and Tunisia, all of which showed evidence of infection in the vector ***Cx*.** *pipiens* s.l.. Countries with no data on study on vectors are colored in gray. Among countries with available data, only Lebanon had zero infection rates for all studied vector species.

Nine studies investigated arthropods in order to analyze the WNV infection rate among vectors. These reports were from Djibouti (n = 2), Egypt (n = 2), Iran (n = 2), Lebanon (n = 1), Pakistan (n = 1), and Tunisia (n = 1). The primary vector of WNV, *i*.*e*., *Cx*. *pipiens* s.l. [2], was detected in Djibouti, Egypt, Iran, and Tunisia, and in all theses countries WNV infection in *Cx*.*pipiens* s.l. was identified. WNV infection was also detected in a wide range of other vector species, including *Cx*. *quinquefasciatus* (Djibouti), *Ae*. *caspius* (Iran), *Cx*. *antennatus* (Egypt), *Cx*. *perexiguus* (Egypt), and *Argas reflexus hermannii* (Egypt). Details for studies on WNV infection vectors are provided in Table 4 and Fig 3.

**Table 4:** Summary of studies on the West Nile virus infection rate in vectors of the EMRO region (n = 9).

Author, Pub. year	Study year	Country	City/governorate	Species	SS	Collection Method	Pools	Test	MIR*	Ref
							(n)		(n/1000)	
**Faulde, 2012**	2010–2012	Djibouti	Djibouti	*Cx*.* quinquefasciatus*	19069	CDC-light Traps	NA	RT-PCR	0.9	[24]
				*Cx*.* pipiens* s.l.	686				2.9	
**Faulde, 2010**	2008–2009	Djibouti	Djibouti	*Culex *spp.	600	CDC- light Traps	NA	RT-PCR	0	[86]
**Turell, 2002**^**§**^	1993	Egypt	Aswan city (3 villages: NagÕ El Hagar, Sabil AbuEl Magd, ElRaghama, NagÕ El Ghuneimiya, El Naghaghra)	Total	36024	NA	32	VI+IF	0.8	[87]
				*An*. *multicolor*	5				0	
				*An*. *pharoensis*	145				0	
				*An*. *tenebrosus*	245				0	
				*Cx*.* antennatus*	2691				1.9	
				*Cx*.* perexiguus*	9011				2.6	
				*Cx*. *pipiens* s.l.	6982				0.3	
				*Cx*. *poicilipes*	26				0	
				*Ae*. *caspius*	16889				0	
				*Uranotaenia unguiculata*	30				0	
				Sand flies	676				0	
				*Culicoides* spp.	200				0	
				Hard ticks	78				0	
**Schmidt 1964**	1960	Egypt	Sheikh	*Argas reflexus hermanni*	1400	NA	28	VI	4.3	[88]
**Bagheri, 2015**	2015	Iran	West Azerbaijan	*An*. *maculipennis*	368	Dipping	45	RT-PCR	0	[15]
				*Cx*.* longiareolata*	130				0	
				*Cx*. *hortensis*	1				0	
				*Cx*.* pipiens* s.l.	354				0	
				*Cx*.* theileri*	618				0	
				*Ae*. *caspius*	672				33.3	
**Shahhosseini, 2017**	2015–2016	Iran	Gilan, Mazandaran, Golestan, East Azerbaijan, Lorestan	*Cx*. *pipiens* s.l.	21060	Biogents Sentinel Traps	1222	RT-PCR	0	[14]
				*Cx*. *Sitiens*	10995				0	
				*Cx*. *theileri*	3856				0	
				*Cx*. *perexiguus*	486				0	
				*Cx*. *pipiens* s.l.	326				3.1	
				*An*. *hyrcanus*	180				0	
				*An*. *maculipennis* s.l.	117				0	
				*An*. *superpictus*	109				0	
				*Cx*.* tritaeniorhynchus*	42				0	
				*An*. *stephensi*	16				0	
				*An*. *claviger*	15				0	
				*Cx*.* pipiens* form *pipiens* x *molestus*	15				0	
				*Culiseta longiareolata*	15				0	
				*Cx*.* mimeticus*	14				0	
				*Cx*.* hortensis*	11				0	
				*Ae*. *caspius*	8				0	
				*An*. *pseudopictus*	8				0	
				*Cx*. *pipiens* cf. *quinquefasciatus*	8				0	
				*An*. *dthali*	6				0	
				*An*. *fluviatilis* s.l.	6				0	
				*Cx*. *pipiens pipiens* form *molestus*	5				0	
				*An*. *apoci*	4				0	
				*An*. *marteri*	4				0	
				*An*. *plumbeus*	4				0	
				*Ae*. *vexans*	2				0	
**Garabedian, 1971**^**ǂ**^	1962–1963	Lebanon	NA	*Aedes* spp. (mostly)	5131	NA	NA	VI	0	[18]
**Reisen, 1982**	1978–1979	Pakistan	Punjab	*Cx*.* tritaeniorhynchus*, *Cx*.*quinquefasciatus*, *Cx*.* pseudovishnui*	44797	Dipping, Biting, Resting outdoors & indoors	NA	VI	0	[89]
**Wasfi, 2016**	2014	Tunisia	El Felta, Saddaguia	*Cx*. *pipiens* s.l.	102	CDC-light Traps	21	RT-PCR	68.6	[90]

* MIR was calculated by dividing the number of positive pools by the total number of specimens tested and multiplied by 1000. Where the number of tested specimens is below 1000, the MIR may not accurately represent the true infection rate in the population, and should be interpreted with caution.

§ Mosquitoes were sorted to species, pooled, and processed for virus isolation both by intracerebral inoculation into suckling mice and by inoculation into cell culture. A total of 33 virus isolates was made from 36,024 mosquitoes. Virus identification was performed using indirect fluorescent antibody testing.

^**ǂ**^ The data for this studiy is driven from articles’ abstract, as their full-text could not be obtained.

Abbreviations: **SS:** Sample Sizes, **MIR:** Minimum Infection Rate, **NA:** Data was not available, **RT-PCR:** Reverse Transcriptase-Polymerase Chain Reaction, **VI:** Virus Isolation, **IF:** ​​Immunofluorescence Assay.

## Discussion

Seroprevalence of WNV has been investigated in 14 of 22 countries in the EMRO region. Since 1942, WNV antibodies have been detected in the general population in 11 countries with available data, including: Djibouti, Egypt, Iran, Iraq, Jordan, Lebanon, Libya, Morocco, Pakistan, Sudan, and Tunisia. Our results also suggested that the overall seroprevalence of WNV has been lower in reports from more recent years (since 2010) compared to reports compiled between 1942 and 2009.

Although the presence of WNV infection remains unknown in countries without data in the EMRO region (n = 14), it can be implied that the virus may probably circulate within these countries as well. Existing evidence suggests cross-country dispersion of a number of viruses such as human immunodeficiency virus (HIV) [91] and hepatitis B virus (HBV) [92]. These observations can imply the hypothesis in which WNV also have dispersed across countries in the region, affecting localities (countries) adjacent to infected areas. The argument is further strengthened if we consider the transmission routes of HIV, HBV, and WNV. The transmission of HIV and HBV depends on effective human-to-human contacts, which acts as a barrier for virus dispersion over large geographic distances. However, similar to other arboviruses like Dengue and Crimean-Congo Hemorrhagic Fever [93, 94], the cross-country spread of WNV can be much easier and fast as it can be transmitted through a broad range of vectors and reservoirs.

Most of the seroepidemiological studies included in this review used ELISA for the detection of anti-WNV antibodies. Although this assay is simple, sensitive, and commercially available, it suffers from cross-reactivity with antibodies raised against other flaviviruses. So, using the ELISA method for testing individuals with a history of vaccination against, or infection with related flaviviruses can yield false positive results [95]. To achieve a more specific measurement, positive ELISA test results should be confirmed by the plaque reduction neutralization test (PRNT), which is considered as the gold standard method for WNV serological testing. However, PRNT can detect antibodies at levels that neutralize the virus; therefore, it has low sensitivity for seroepidemiological studies in weakly-exposed populations [95].

Approximately, one-fifth of WNV infected individuals demonstrate symptomatic infection [96]. Clinical symptoms are also non-specific to the disease and include fever, malaise, headache, back pain, myalgia, and anorexia. Therefore, WNV infected individuals can be misdiagnosed with other febrile infections. In areas with evidence of WNV circulation, WNV infection should be considered as a differential diagnosis for patients demonstrating non-differential febrile syndroms.

Non-specific sympotoms of the WNV infection also highlights the need for laboratory testing of suspected human cases. While WNV IgM is the most common target for confirmation of the infection, viral RNA testing can also be performed. Combining IgM detection and viral RNA testing can enhance the possibility of diagnosis in patints with West Nile fever, as indicated by Tilley et al. [97]. However, among 15 studies on patient populations, only four used a combination of serological and molecular assays for the diagnosis of WNV infection.

In this review, we have highlighted serological evidence of WNV infection from 22 independent studies conducted on animal populations in the region. These studies were carried out in 10 countries including, Djibouti, Iran, Jordan, Morocco, Pakistan, Palestine, Qatar, Saudi Arabia, Tunisia, and the UAE. Most studies, have investigated evidence of WNV infection among domestic animals. Since 2010, the highest prevalence of WNV among domestic animals, has been reported among dogs of Morocco and equids of Morocco, Pakistan, Palestine and Iran. The high rates of animal seropositivity and geographic distribution of animal infection reflect the favorable conditions for the circulation of WNV in these countries. In these areas, stronger preventive measures should be considered to reduce the risk of WNV transmission to humans and horses. High seropositivity among dogs and equids also suggests that these animals can be useful sentinels for WNV surveillance, as discussed by previous studies [98–100]. Resnick, et al. (2008) reported that WNV seroconversion in dogs happened six weeks prior to the infection in exposed human cases [100].

Only two studies from Pakistan (on rodents) [45] and Djibouti (on wild ruminants) [66]) have investigated wild animals’ infection with WNV. The paucity of published studies on the prevalence of WNV infection in wild animals of the EMRO region underlines a gap in current knowledge about the issue. Knowledge about the reservoirs’ infection and virus circulation among wild animals has important implications for forecasting the emergence or re-emergence of WNV epidemics[95]. So, it is recommended future seroprevalence studies include representative samples from wild animals to further illuminate the state of the infection among these hosts.

Four studies investigated the infection among birds from Iran, Morocco, and Tunisia, from which only two studies were recently performed (*i*.*e*., Tunisia, 2015 and 2017). These observations also highlight a gap in current knowledge, this time, on the extend of the infection among birds of the EMRO region. Birds play a critical role in the maintenance and spread of the virus. Prolonged high levels of viremia have been demonstrated in several bird species [101, 102]. The virus has also been isolated from several migratory birds. Thus, surveillance of WNV infection among birds would be of great importance, especially in areas with favorable ecological conditions for birds and mosquitoes. In this regards, a better understanding of birds migration routes would be helpful in selecting the most probable sites for tracking the virus [102], and subsequently making judgments on what areas might be focal points for the emergence of WNV outbreaks.

Mosquitoes and birds are currently considered to have the key role in the life cycle of the virus [2]. However, there are more than 30 other vertebrates such as lemurs, frogs, hamsters, squirrels, rabbits, and chipmunks that have been reported as possible reservoirs for the virus, since they can provide viremia levels that are sufficient to infect mosquito vectors [103]. The role of these reservoirs in the WNV life cycle and epidemic has been less regarded till now, and is an open area for future research.

Despite the critical role of the vector in the life cycle and the epidemic of WNV, only nine studies have investigated vector infection in the region. These studies have been conducted in Djibouti, Egypt, Iran, Pakistan, and Tunisia. The primary vector of WNV, *i*.*e*., *Cx*. *pipiens* s.l. [2] was detected in all investigared countries except Pakistan. Although WNV infection has been detected in more than 60 mosquito species, detection of viral infection in a mosquito alone does not indicate that the mosquito is a competent vector for the virus. In addition to *Culex* species, WNV has also been detected in *Aedes* and *Mansonia* mosquitoes. Additional studies are necessary to further clarify the potential role these species in the maintenance and transmission of WNV. Interestingly, WNV infection was observed in ticks *Argas reflexus hermannii*. Previous studies from other regions of WHO also detected WNV RNA in ticks *R*. *turanicus* and *mites D*. *gallinae* and *O*. *sylvarum*. However, their competency as vectors is less clear [104]. Reducing virus transmission from a vector is one of the main strategies of controlling arboviral diseases. Therefore, more efforts to identify the main vectors and understand virus–vector interaction in burdened countries would benefit disease control strategies [105].

The main limitations of this systematic review relate to the data. First, there is a paucity of prevalence studies in the EMRO region, and the quality of data reported by studies varied. For instance, many available studies on human populations were focused on adults, or did not report age and gender for the study sample. The remaining studies included a broad range of age groups (including infant, children, and adults), most of which did not report age and gender specific prevalence. Prevalence data on healthy infants and young children alone was particularly sparse. Therefore, the state of the epidemic among different age and sex groups remains unknown in this region and requires further study with representative samples. Although current data provides a good basis for an overall judgment about the presence of current/past WNV exposure in most investigated samples, they can hardly be used to infer the actual prevalence and state of the epidemic in most investigated countries. For example, only in four countries with available data on the ‘general population’ (*i*.*e*., Egypt, Iran, Lebanon, and Pakistan), the total number of tested individuals was reasonably representative of the target population (*i*.*e*., more than 1000). These ‘powerful’ studies, however, were not totally flawless. One of the main limitations of these studies was that some of them had used convenience (non-random) sampling methods. In convenience sampling, individuals have unequal and unknown probability of being selected [106]. Hence, the resulting seroprevalence estimates should be generalized to the target population with caution. Few studies available from animal populations in the region also suffered from the abovementioned shortcomings; *i*.*e*., non-random sampling and small sample sizes. For example, the seroprevalence of WNV has been investigated in Morocco, Palestine, and Tunisia, but only the study in Morocco has provided the estimate based on a fairly representative sample of 556 and 836 camels for the years 2003 and 2009, respectively. The case was even worse for the seroprevalence studies on dogs, cows, sheep, goats, buffalos, and birds as none of the available studies were well-powered enough (*i*.*e*., had small sample sizes). The situation was more satisfactory for the population of horses, where a number of studies with large sample sizes were available from different parts of Iran, Morocco, Pakistan, Palestine, Tunisia, and the UAE. Second, the relative dearth of recent seroprevalence studies, particularly from burdened areas for WNV infection and high-risk population groups is a serious limitation. As the face of WNV disease and its geographic range changes rapidly, WNV prevalence estimated by older studies may not properly reflect the current status of WNV circulation. Less accurate serological tests used by older studies also affect the validity and reliability of the prevalence estimates in these studies. Standardized seroprevalence studies at national levels are critical to best appraise the epidemic status, the impact of interventions and the potentials for future outbreaks. Third, substantial within-country heterogeneity in the prevalence of WNV was noted. This might be due to diversity in the geographical areas, target groups, and the reported sample sizes of studies. Local prevalence estimates, hence, might not be representative of national level prevalence, particularly in large countries with much geographic and ethnic disparities. Finally, our review is limited to reports written in English.

## Conclusions

This review provides estimates of the scale of the WNV epidemic at country and regional levels in order to inform efforts for developing and implementing effective future responses. Our results suggested the circulation of WNV in humns, animals, or vectors of most investigated countries in the region. However, there is paucity of data about WNV infection, especially with respect to the burden of the infection in most countries across the region. Hence, further epidemiological studies that take into account the human, reservoir and vector dimension/aspect of the occurrence and distribution of the virus should be conducted particularly in high-prevalent countries. Such research effort will generate robust knowledge and a detailed understanding of the epidemiology of the infection in local populations, and foster in-depth investigations about transmission patterns of the virus. Identification of the geographic distribution of primary reservoirs of the virus and their infection status can also enhance targeted prevention and elimination efforts and aid forecasting attempts. Moreover, surveillance capacities in EMRO countries ought to be established or expanded for better monitoring of WNV infection at national and regional levels.

## Supporting information

S1 File PRISMA 2009 checklist. (DOC)

S2 File Search strategy. (DOCX)
