# Supplementary material for: Epidemiology of West Nile Virus in the Eastern Mediterranean region: A systematic review
Source: PLoS Negl Trop Dis. 2019 Jan 29;13(1):e0007081. doi: 10.1371/journal.pntd.0007081 (PMC6368338; doi:10.1371/journal.pntd.0007081)
Supplement: S2 File — (DOCX) [file pntd.0007081.s002.docx]

| **WNV in the EMRO Search Strategy** |
| --- |
| **Last search** |
| - Jan 30, 2018 |
| **Databases** |
| 1. PubMed |
| 1. Scopus |
| 1. Web of Science |
| 1. Google Scholar |
| 1. Index Medicus for the Eastern Mediterranean Region (IMEMR) |

**Search strategy for databases 1 to 3:**

((((WNV) OR "West Nile Virus") OR "West Nile fever")) AND (("EMRO") OR "Eastern Mediterranean Region")

OR

((((WNV) OR "West Nile Virus") OR "West Nile fever")) AND (("Afghanistan") OR " Afghan*")

OR

((((WNV) OR "West Nile Virus") OR "West Nile fever")) AND Bahrain*

OR

((((WNV) OR "West Nile Virus") OR "West Nile fever")) AND Djibouti*

OR

((((WNV) OR "West Nile Virus") OR "West Nile fever")) AND Egypt*

OR

((((WNV) OR "West Nile Virus") OR "West Nile fever")) AND Iran*

OR

((((WNV) OR "West Nile Virus") OR "West Nile fever")) AND Iraq*

OR

((((WNV) OR "West Nile Virus") OR "West Nile fever")) AND Jordan*

OR

((((WNV) OR "West Nile Virus") OR "West Nile fever")) AND Kuwait*

OR

((((WNV) OR "West Nile Virus") OR "West Nile fever")) AND Lebanon

OR

((((WNV) OR "West Nile Virus") OR "West Nile fever")) AND Lebanese

OR

((((WNV) OR "West Nile Virus") OR "West Nile fever")) AND Libya*

OR

((((WNV) OR "West Nile Virus") OR "West Nile fever")) AND Morocco

OR

((((WNV) OR "West Nile Virus") OR "West Nile fever")) AND Moroccan

OR

((((WNV) OR "West Nile Virus") OR "West Nile fever")) AND Oman*

OR

((((WNV) OR "West Nile Virus") OR "West Nile fever")) AND Pakistan*

OR

((((WNV) OR "West Nile Virus") OR "West Nile fever")) AND Palestine*

OR

((((WNV) OR "West Nile Virus") OR "West Nile fever")) AND Qatar*

OR

((((WNV) OR "West Nile Virus") OR "West Nile fever")) AND “Saudi Arabia*”

OR

((((WNV) OR "West Nile Virus") OR "West Nile fever")) AND Somalia*

OR

((((WNV) OR "West Nile Virus") OR "West Nile fever")) AND Sudan*

OR

((((WNV) OR "West Nile Virus") OR "West Nile fever")) AND “Syrian Arab Republic”

OR

((((WNV) OR "West Nile Virus") OR "West Nile fever")) AND Syria*

OR

((((WNV) OR "West Nile Virus") OR "West Nile fever")) AND Tunisia*

OR

((((WNV) OR "West Nile Virus") OR "West Nile fever")) AND “United Arab Emirates”

OR

((((WNV) OR "West Nile Virus") OR "West Nile fever")) AND Yemen*

**Search strategy for Google Scholar:**

((((WNV) OR "West Nile Virus") OR "West Nile fever") AND ("EMRO" OR "Eastern Mediterranean Region" OR "Afghanistan" OR " Afghan*" OR Bahrain* OR Djibouti* OR Egypt* OR Iran* OR Iraq* OR Jordan* OR Kuwait* OR Lebanon OR Lebanese OR Libya* OR Morocco OR Moroccan OR Oman* OR Pakistan* OR Palestine OR Qatar* OR “Saudi Arabia*” OR Somalia* OR Sudan* OR Syria* OR Tunisia* OR “United Arab Emirates” OR Yemen*))))

**Search strategy for Index Medicus for the Eastern Mediterranean Region (IMEMR):**

A search was performed by the keywords: ((((WNV) OR "West Nile Virus") OR "West Nile fever"))

**Hand searching:**

Reference list of included articles were searched to find additional citations not found in database searches.
